# Synergistic Hydrogels Enabled by Dual-Regulatory Mussel Foot Protein for Advancing Wound Healing

**DOI:** 10.3390/gels12070627

**Published:** 2026-07-14

**Authors:** Jiren Xu, Na Li, Chen Wang, Jeevithan Elango, Wenhui Wu, Peng Fu, Bailei Li

**Affiliations:** 1 Department of Marine Pharmacology, College of Food Science and Technology, Shanghai Ocean University, Shanghai 201306, China; m240451270@st.shou.edu.cn (J.X.); d220300085@st.shou.edu.cn (N.L.); 15537768768@163.com (C.W.); whwu@shou.edu.cn (W.W.); 2 Department of Biomaterials Engineering, Faculty of Health Sciences, UCAM-Universidad Católica San Antonio de Murcia, Campus de los Jerónimos 135, Guadalupe, 30107 Murcia, Spain; jelango@ucam.edu; 3 Center of Molecular Medicine and Diagnostics (COMManD), Department of Biochemistry, Saveetha Institute of Medical and Technical Sciences, Saveetha Dental College and Hospitals, Saveetha University, Chennai 600077, India; 4 Putuo Branch of International Combined Research Center for Marine Biological Sciences, Zhoushan 316104, China; 5 Marine Biomedical Science and Technology Innovation Platform of Lin-gang Special Area, Shanghai 201306, China; 6 College of Foreign Languages, Ningbo University of Finance & Economics, Ningbo 315175, China; 7 Department of Biotechnology and Biomedicine, Zhejiang Key Laboratory of Multiomics and Molecular Enzymology, Yangtze Delta Region Institute of Tsinghua University (Zhejiang), Jiaxing 314006, China

**Keywords:** mussel foot protein, network-regulated hydrogel, β-chitosan, sodium hyaluronate, wound healing

## Abstract

Impaired wound healing is often caused by persistent inflammation, bacterial infection, and insufficient extracellular matrix remodeling. Natural polymer-based hydrogels represent ideal wound dressings but often struggle to balance structural stability and biological activity. Herein, we report a dual-functional network regulation strategy enabled by highly soluble mussel foot protein (HMFP) that acts simultaneously as a structural crosslinking regulator and bioactive effector to fabricate synergistic hydrogels (CS-SH-H) from β-chitosan (CS) and sodium hyaluronate (SH). HMFP homogenizes the porous microstructure, strengthens intermolecular interactions, and significantly improves thermal and structural stability via multivalent non-covalent bonding. In vitro, CS-SH-H shows excellent cytocompatibility, significantly promotes fibroblast proliferation and migration, and exerts potent antibacterial activity against *Escherichia coli* (*E. coli*) and *Staphylococcus aureus* (*S. aureus*). In a mouse full-thickness skin defect model, the hydrogel dramatically accelerates wound closure, reducing the residual wound area to 25% on day 7, outperforming the control groups. Immunohistochemistry confirms that HMFP suppresses TNF-α-mediated inflammation and enhances Ki-67-positive cell proliferation, leading to accelerated re-epithelialization and collagen deposition. This study establishes HMFP as a promising marine-derived dual-functional network regulator for designing high-performance hydrogel dressings. This strategy is scalable and translatable for treating infected and inflammatory wounds.

## 1. Introduction

Chronic and complex skin wound healing, an intricate biological cascade, is often critically impaired by sustained inflammation, bacterial infection, and insufficient extracellular matrix (ECM) remodeling [1,2,3]. Polymeric hydrogels, owing to their high water content and tunable three-dimensional (3D) architecture that mimics the native ECM, have emerged as promising therapeutic dressings to accelerate this process [4,5,6]. Among them, hydrogels derived from natural polysaccharides offer inherent biocompatibility, biodegradability, and low immunogenicity [7,8,9]. However, this category of biomaterials faces a central and persistent dilemma in achieving both robust structural resilience under physiological shear and high, multifaceted bioactivity, a trade-off that fundamentally limits their efficacy in the dynamic, complex milieu of non-healing wounds [10,11,12]. While capable of providing a basic hydrated scaffold, polysaccharide-based hydrogels frequently exhibit compromised mechanical integrity, limited functional versatility, and a lack of dynamic regulation over inflammatory cascades and cell behaviors, which are crucial for orchestrating a complete regenerative response.

Conferring additional functionalities to these polysaccharide networks typically involves incorporating bioactive agents. In this regard, mussel adhesive proteins (MAPs) have attracted immense interest as a source of bio-inspiration [13,14,15]. These properties have propelled MAPs beyond mere adhesive applications into the realm of functional hydrogel fabrication [16,17,18]. Beyond their classical role in adhesion, MAP-derived components, through their unique catechol (e.g., DOPA) and amine chemistries, have been utilized as functional additives to enhance various hydrogel properties, including cohesion, mechanical robustness, and bio-adhesiveness. The emerging vision is to elevate MAPs from simple ‘bioactive dopants’ to sophisticated ‘network regulators’ that can actively guide hydrogel self-assembly and impart multifunctionality [19,20,21]. Unfortunately, this transformative potential is severely hampered by a fundamental material-level constraint: the poor aqueous solubility and tendency to aggregate of conventionally processed MAPs [22,23,24]. This material-level deficiency not only hinders their uniform and stable integration into hydrogel matrices but, more importantly, prevents them from acting as dynamic, site-specific crosslinkers or structural organizers. Consequently, they are often trapped in a passive, diffusible ‘cargo’ role rather than performing as integral network regulators [25].

Collagen, gelatin, and bovine serum albumin are widely used materials in protein-based wound dressings; however, they typically serve only as passive scaffolds or sources of a single bioactive factor, and it is often difficult to achieve both structural support and functional regulation simultaneously [26,27,28]. MAPs exhibit excellent interfacial adhesion and bioactivity due to their unique catechol chemistry [29,30,31]. However, traditional MAP has poor water solubility and is prone to aggregation, making it difficult to disperse uniformly within a gel network. Furthermore, it cannot actively participate in the cross-linking process and is therefore mostly limited to playing a passive role as a bioactive factor [32]. To transcend this long-standing solubility-structure- function barrier, we previously engineered a novel highly soluble mussel foot protein (HMFP) with a molecular weight primarily in the 11–17 kDa range while preserving its key functional motifs such as catechol and amine residues [33]. Building on this breakthrough, we hypothesize that this highly soluble HMFP can act as a dual-functional ‘network regulator’ within a polysaccharide hydrogel matrix. We propose that HMFP actively participates in and spatially guides the self-assembly of hydrogel networks, leveraging its multivalent interactions to regulate network organization and enhance structural stability. Critically, this structural role is intrinsically coupled with its bioactive effector functions, aiming to create a synergistic platform where the mechanical and biological enhancements arise from the same molecular component, co-reinforcing each other.

To demonstrate this paradigm, we constructed a composite hydrogel system (termed CS-SH-H) using β-chitosan (CS) and sodium hyaluronate (SH), both known for their wound-healing benefits [34,35,36,37]. The CS/SH matrix is primarily held together by electrostatic and hydrogen-bonding interactions, forming a physically crosslinked base network. Herein, we introduce HMFP into this base to act as a dynamic crosslinker and modulator. The abundance of functional groups on HMFP is postulated to form multi-point, non-covalent bridges (e.g., hydrogen bonds, ionic, and hydrophobic interactions) between CS and SH chains, thereby homogenizing the network architecture, reinforcing its structural integrity, and enhancing physicochemical stability [38,39,40]. Concurrently, this integrated structure is designed to sustainably deliver HMFP’s intrinsic pro-healing, anti-inflammatory, and antibacterial bioactivities. In this study, we systematically investigate the multi-scale impact of HMFP incorporation, from microstructural reorganization and enhanced thermal stability to in vitro cell proliferation, antibacterial activity, and ultimately in vivo full-thickness wound healing efficacy. We demonstrate that the HMFP-mediated network regulation effectively coordinates the inflammation-proliferation cascade, validating our strategy for designing the next generation of high-performance, biomimetic wound dressings.

## 2. Results and Discussion

### 2.1. HMFP Acts as a Regulator of Network Organization and Structural Stabilization, Homogeneously Regulating Hydrogel Microstructure and Stability

**Figure 1:**
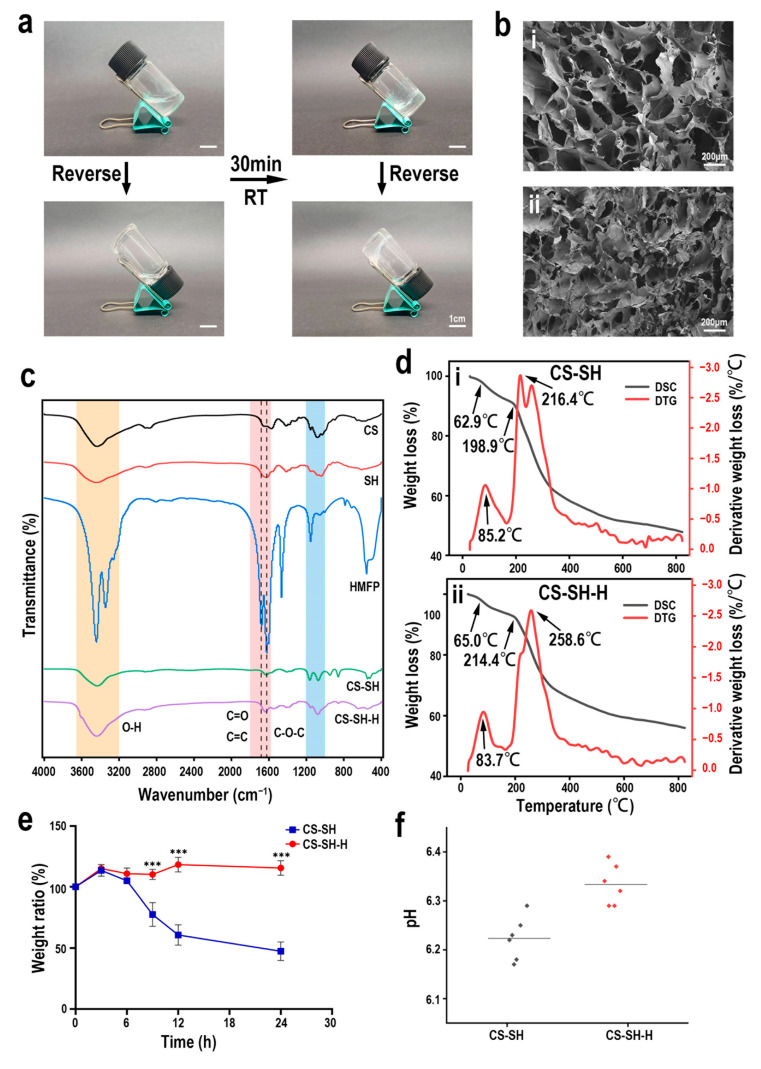
Fabrication, microstructure, and physicochemical characterization for CS-SH and CS-SH-H hydrogels. (**a**) Schematic diagram of hydrogel formation via electrostatic interactions between β-chitosan and sodium hyaluronate. (**b**) SEM images of freeze-dried CS-SH (**i**) and CS-SH-H (**ii**) hydrogels, showing three-dimensional porous structures and surface morphology differences (scale bar: 100 μm). (**c**) FTIR spectra of freeze-dried CS-SH and CS-SH-H hydrogels, illustrating changes in intermolecular interactions after HMFP incorporation. (**d**) TGA and derivative DTG curves of CS-SH (**i**) and CS-SH-H (**ii**) hydrogels under a nitrogen atmosphere, used to evaluate thermal stability. (**e**) Stability profile of CS-SH and CS-SH-H hydrogel in PBS solution (*n* = 3). (**f**) pH values of extracts from CS-SH and CS-SH-H hydrogels (*n* = 6). Abbreviations: differential scanning calorimetry curve (DSC); derivative thermogravimetric curve (DTG); chitosan (CS); sodium hyaluronate (SH); CS-SH hydrogel (CS-SH); CS-SH hydrogel containing 0.1 mg/mL HMFP (CS-SH-H). *** *p* < 0.001.

Given that HMFP has been shown to possess anti-inflammatory and antioxidant activity in our previous studies, we next investigated its properties when incorporated into the β-chitosan/sodium hyaluronate (CS-SH) hydrogel, forming the composite designated CS-SH-H [33,41]. The gel state of the hydrogels was first confirmed by a vial inversion test, in which the CS-SH and CS-SH-H samples exhibited sol-to-gel transition and remained stable without flow upon inversion, indicating the formation of a self-supporting three-dimensional network (Figure 1a). In addition, the ratio of β-chitosan to sodium hyaluronate was optimized through preliminary formulation screening experiments. Based on a reported reference ratio (1:1.8–2.2) [42], three formulations (1:1, 1:2, and 1:3) were evaluated. The 1:1 formulation resulted in an opaque system with visible precipitation, while the 1:3 formulation failed to form a stable hydrogel and remained in a fluid state. In contrast, the 1:2 formulation produced a homogeneous, transparent, and stable hydrogel with desirable gelation behavior and was therefore selected for subsequent experiments. The microstructure, analyzed by scanning electron microscopy (SEM), revealed a profound transformation upon HMFP integration. In contrast to the irregular and heterogeneous porous network of the base CS-SH hydrogel, the CS-SH-H hydrogel exhibited a significantly refined and more homogeneous morphology, characterized by densely interconnected and uniformly distributed pores (Figure 1b). This structural remodeling suggests that HMFP does not act as a passive filler but actively directs polysaccharide self-assembly, thereby modulating the overall network architecture.

Molecular-level evidence from attenuated total reflection Fourier-transform infrared (FTIR) spectroscopy supports this structural regulation. The CS-SH-H spectrum displayed a noticeable enhancement and broadening of the broad band in the 3200–3700 cm^−1^ region (characteristic of O-H and N-H stretching vibrations) (Figure 1c), suggesting a significant increase in overall hydrogen bonding density after HMFP incorporation. Additionally, simultaneous spectral variations in the 1600–1800 cm^−1^ (C=O and C=C stretching vibrations) and 1000–1200 cm^−1^ regions (C-O-C stretching vibrations), and subtle yet distinct changes in the amide I (1640–1660 cm^−1^) region confirm the successful and interactive integration of HMFP protein motifs within the polysaccharide matrix.

Importantly, these microstructural and molecular alterations directly translated to enhanced material robustness. Thermogravimetric analysis (TGA) demonstrated a substantial improvement in the thermal stability of the CS-SH-H hydrogel (Figure 1d). Specifically, the maximum decomposition rate (Td_max), identified from derivative thermogravimetric (DTG) analysis, increased from 216.4 °C for the CS-SH hydrogel to 258.6 °C for the CS-SH-H hydrogel (Figure 1e). Simultaneously, the physiological and chemical compatibility of the modified scaffold was maintained. The degradation/re-swelling curves of the CS-SH-H hydrogel group over 12–24 h in PBS were more stable than those of the CS-SH hydrogel group, and the pH of their extracts remained within a tissue-friendly range (approximately 6.3) (Figure 1f). Collectively, these findings demonstrate that HMFP functions as a regulator of network organization and structural stabilization, enhancing the structural homogeneity and thermal resilience of the hydrogel network while preserving its physical suitability as a wound dressing.

At the material science level, our work re-conceptualizes the role of marine-derived proteins in hydrogel construction. Most reported strategies involving marine polypeptides such as mussel-inspired catecholamines or single-domain adhesives treat them primarily as bioactive additives or secondary crosslinkers to introduce new functional motifs into an otherwise pre-formed and dominant polymer network. While these methods enhance properties like adhesion or toughness, they frequently leave the primary network’s morphology and inherent weaknesses largely unaddressed [43]. This study establishes a critical departure: by employing a highly soluble HMFP variant that is functionally integrated before network assembly (co-gelation) rather than being simply “stirred-in” or tethered post-gelation, it serves as a potent intrinsic structural modifier. This is distinct from passive fillers, which typically remain as distinct phases or aggregates within the pores [17,44,45]. Crucially, the consequent dramatic enhancement in stability (Figure 1d,e) directly reflects an overall supramolecular network reinforcement effect. This is ascribed to HMFP’s amphiphilic nature and the abundant DOPA, amino, and hydroxyl residues, which allow it to form multivalent, non-covalent cross-bridges (including hydrogen bonds, electrostatic, and hydrophobic interactions) between the polysaccharide chains.

### 2.2. HMFP Exhibits Concentration-Dependent Pro-Activity and Biosafety Towards Fibroblasts

**Figure 2:**
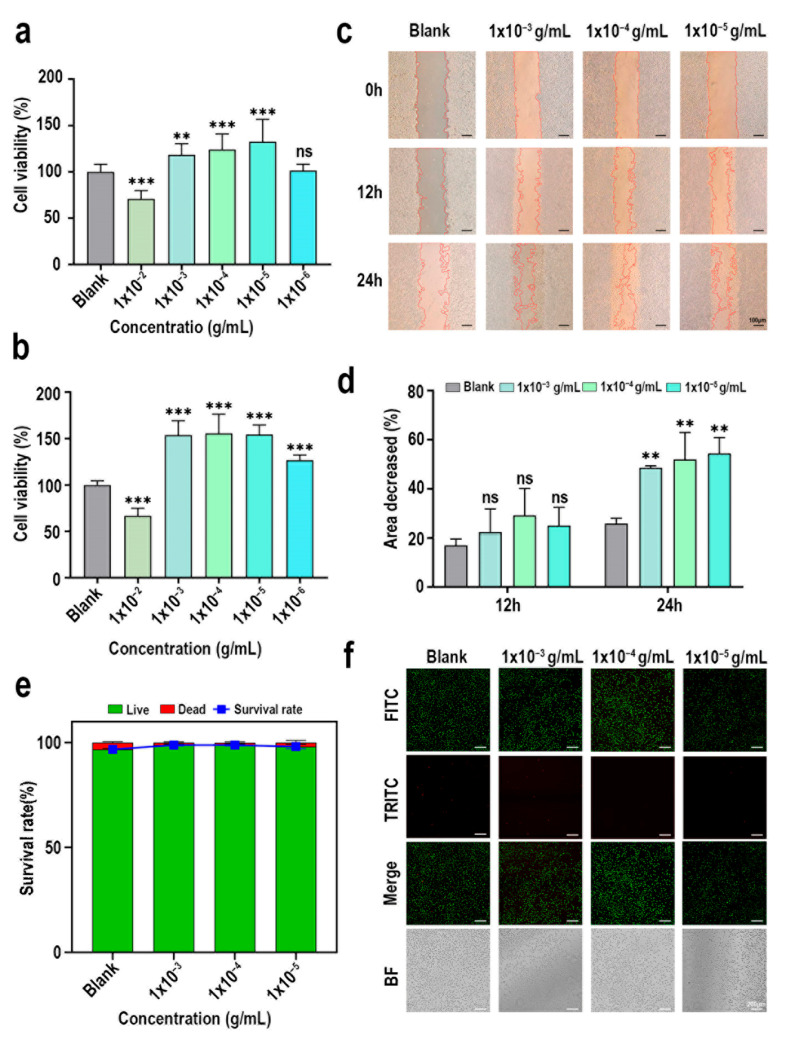
Effects of HMFP on proliferation, migration, and viability of L929 fibroblasts. Cell proliferation of L929 cells after treatment with different concentrations of 1 × 10^−2^–1 × 10^−6^ g/mL HMFP for 24 h (**a**) and 48 h (**b**) using the CCK-8 assay (*n* = 6). (**c**,**d**) Scratch wound-healing assay of L929 cells following HMFP treatment for 12 and 24 h, showing representative images (scale bar: 100 μm) and quantitative analysis of wound area reduction (*n* = 3). (**e**,**f**) Live/dead staining of L929 cells after 24 h HMFP treatment assessed by Calcein-AM/PI staining, showing representative fluorescence images (scale bar: 200 μm) and quantitative analysis of live/dead cell ratios. Group: fluorescently labeled live cells (FITC); fluorescently labeled dead cells (TRITC); FITC+TRITC (Merge); brightfield (BF). ** *p* < 0.01, *** *p* < 0.001, ns denotes no significant difference.

To establish a foundation for its integration, the intrinsic bioactivity and safety profile of HMFP were first evaluated independently. CCK-8 assays revealed that HMFP exerts a significant and concentration-dependent regulatory effect on L929 fibroblast viability. While a high concentration (1 × 10^−2^ g/mL) significantly inhibited cell growth, optimal concentrations (1 × 10^−3^–1 × 10^−5^ g/mL) markedly promoted proliferation, increasing cell viability by over 15% at 24 h and over 50% at 48 h compared to the untreated control group (Figure 2a,b). Supporting this finding, the HMFP-enhanced proliferative effect was accompanied by accelerated cellular migration in a scratch wound healing assay. Treatment with HMFP (1 × 10^−3^ to 1 × 10^−5^ g/mL) led to approximately 50% wound closure within 24 h, a rate nearly doubling that of the untreated control (approximately 25% closure) (Figure 2c,d). Crucially, no cytotoxicity was observed within this therapeutically effective range. Live/Dead staining confirmed that HMFP treatment at concentrations between 1 × 10^−3^ to 1 × 10^−5^ g/mL sustained exceptionally high fibroblast viability (>98%) after 24 h (Figure 2e,f). These data collectively demonstrate that HMFP is a concentration-tunable bioactive peptide that simultaneously promotes fibroblast proliferation and migration within a well-defined safety window, with optimal effects observed at a concentration of 1 × 10^−4^, providing a clear functional rationale for its incorporation into the hydrogel scaffold and for subsequent experiments.

### 2.3. The Composite Hydrogel Retains Antibacterial Properties and Exhibits Enhanced Cytocompatibility

**Figure 3:**
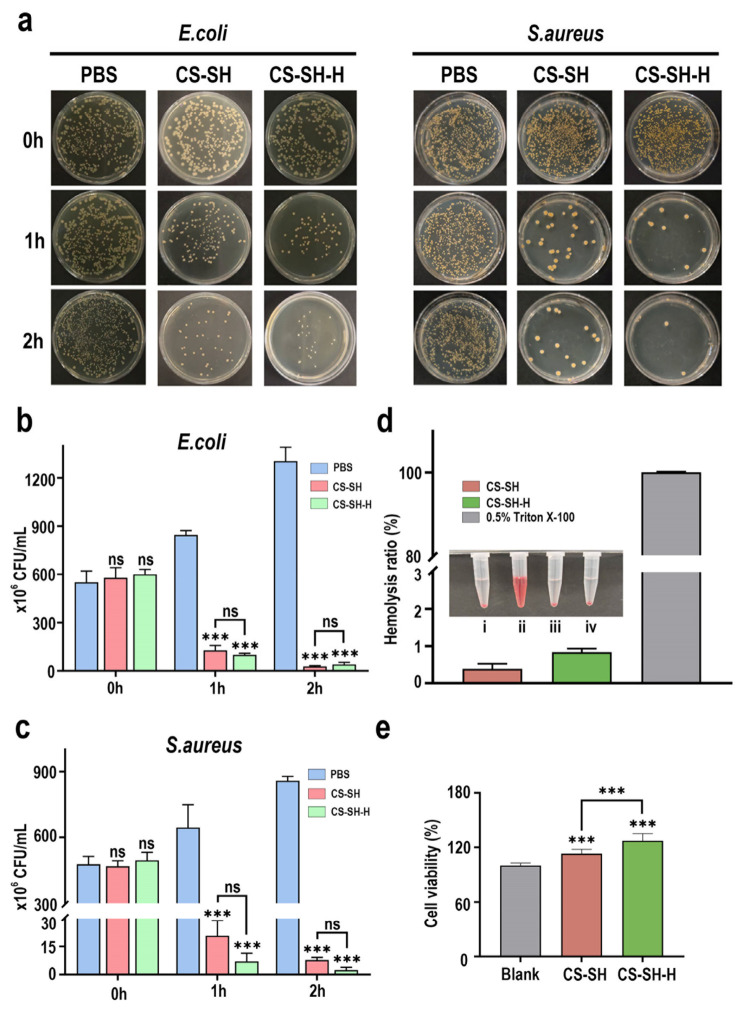
In vitro antibacterial activity, biosafety, and extract-mediated cellular responses of CS-SH and CS-SH-H hydrogels. (**a**) Representative antibacterial images of CS-SH and CS-SH-H hydrogels against *E. coli* and *S. aureus*. (**b**) Quantitative analysis of antibacterial inhibition rates for *E. coli.* (*n* = 3) (**c**) Quantitative analysis of antibacterial inhibition rates for *S. aureus.* (*n* = 3) (**d**) Hemolysis assay of CS-SH and CS-SH-H hydrogels showing representative images and quantitative hemolysis ratios. (*n* = 3) Treatment groups: i, PBS; ii, 0.5% Triton X-100; iii, CS-SH; iv, CS-SH-H. (**e**) Cell viability of L929 fibroblasts treated with 100% hydrogel extracts evaluated by CCK-8 assay (*n* = 6). *** *p* < 0.001, ns denotes no significant difference.

We next evaluated whether the structural optimization compromised the intrinsic functionalities of the hydrogel platform. The fundamental antibacterial activity of the cationic chitosan component was robustly preserved upon HMFP integration. Both CS-SH and CS-SH-H hydrogels achieved potent bactericidal effects against both *E.coli* and *S.aureus*, with inhibition rates exceeding 97% within 2 h of contact (Figure 3a–c), attributed to chitosan’s interaction with bacterial membranes [46,47]. This indicates that the presence of HMFP does not interfere with the electrostatic disruption of bacterial membranes by CS. Excellent blood compatibility was also maintained, as reflected by hemolysis rates (<3%) far below the 5% threshold for biomaterials (Figure 3d), confirming the overall biosafety of the composite formulation.

Beyond maintaining baseline properties, HMFP incorporation conferred enhanced cytocompatibility in a host-directed manner. Cell viability assays revealed that treatment with extracts from the base CS-SH hydrogel was biocompatible, supporting over 90% fibroblast viability (Figure 3e). Strikingly, extracts from the HMFP-incorporated CS-SH-H hydrogel further and significantly elevated this value to approximately 120%. This suggests that the structural integration of HMFP may facilitate a more sustainable and bioavailable release profile of its intrinsic pro-regenerative cues, in contrast to the potential diffusion-limited or conformationally restricted release from simple blends.

Moreover, in our direct cell assays (Figure 2), the optimal concentration range of free HMFP significantly boosted fibroblast proliferation and migration without cytotoxicity. This synergistic in vitro benefit (chitosan’s antibacterial protection and HMFP’s pro-healing promotion), derived from an optimized material network rather than mere drug loading, constitutes the functional cornerstone for enhanced wound repair.

### 2.4. Accelerated In Vivo Wound Healing Mediated by HMFP-Modified Hydrogel

**Figure 4:**
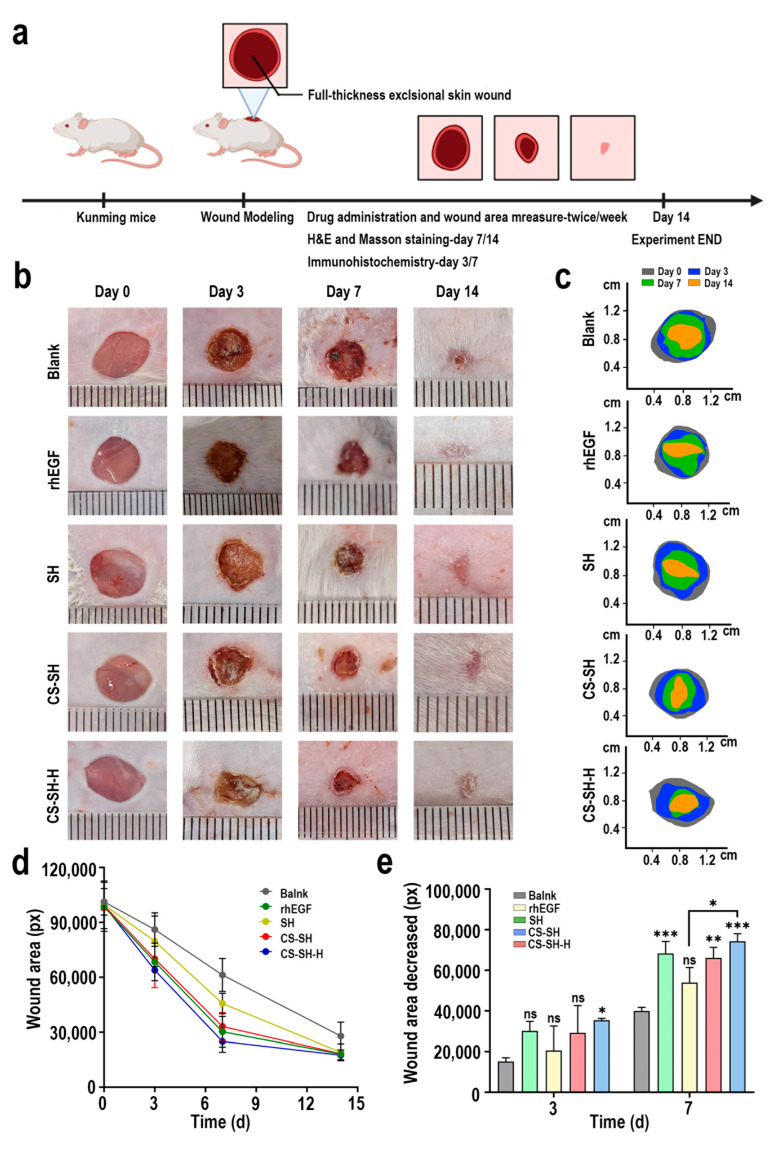
Wound closure performance of hydrogels in a mouse full-thickness skin defect model. (**a**) Schematic illustration of the mouse full-thickness skin defect model and treatment procedure. (**b**) Representative macroscopic images of wounds at days 0, 3, 7, and 14 after initial treatment. (**c**) Schematic illustration of the wound healing process. (**d**) Quantitative analysis of wound areas at different time points for each treatment group (*n* = 3). (**e**) Quantitative analysis of wound area reduction at days 3 and 7 after treatment (*n* = 3). * *p* < 0.05, ** *p* < 0.01, *** *p* < 0.001, ns denotes no significant difference.

The ultimate goal of developing a ‘network-regulated’ strategy was to translate enhanced structural and in vitro properties into superior wound-healing performance. The combined benefits of structural stability and enhanced bioactivity were ultimately assessed in a full-thickness skin defect model in mice (Figure 4a). The CS-SH-H hydrogel demonstrated markedly superior wound closure kinetics throughout the 14-day experiment compared to all control groups, including the base CS-SH hydrogel and the positive control (recombinant human epidermal growth factor, rhEGF) (Figure 4b–d). A critical advantage of the CS-SH-H treatment became evident in the early inflammatory/proliferative phase. By Day 7, wounds treated with CS-SH-H hydrogel achieved 75% area reduction, which was significantly more effective than the CS-SH treatment (67% area reduction) and the positive rhEGF control (70% area reduction) (Figure 4e). Visual assessment confirmed that by Day 14, the CS-SH-H group had achieved nearly complete and aesthetically superior re-epithelialization (Figure 4b). This superior performance can be attributed to a microenvironment sculpted by the HMFP-integrated network.

### 2.5. HMFP Hydrogel Orchestrates Inflammation-Proliferation Balance and Improves Collagen Maturation

**Figure 5:**
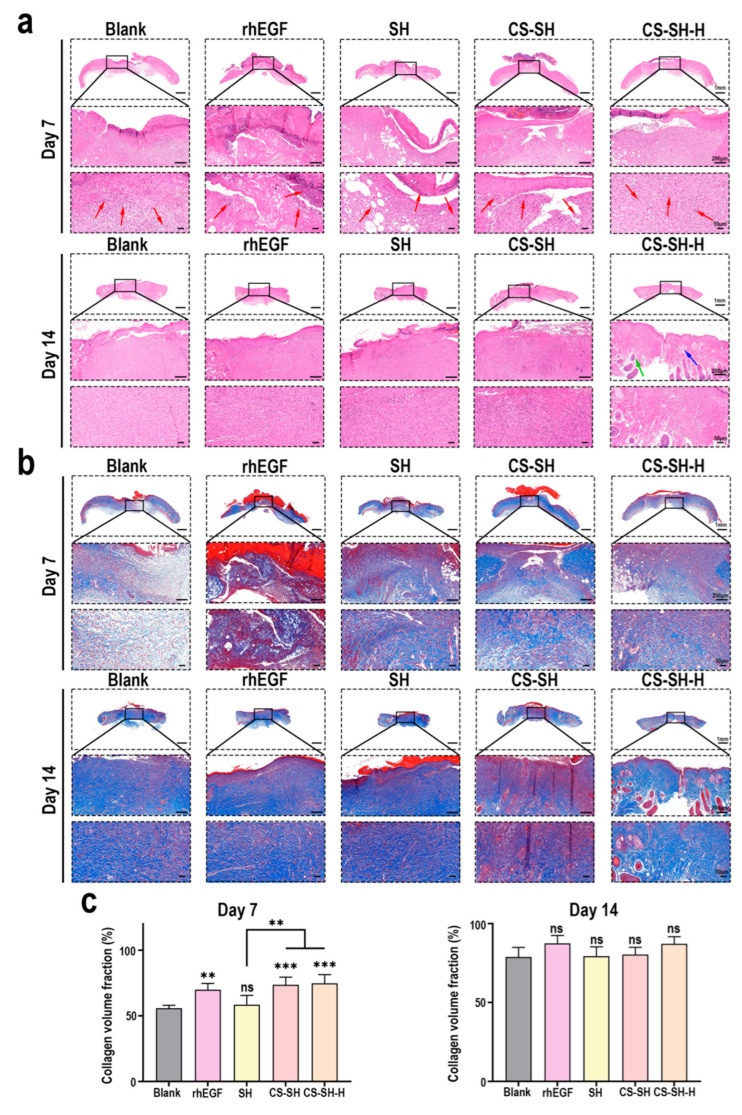
Histological evaluation of wound tissues in different treatment groups. (**a**) Representative H&E staining images of wound tissues at days 7 and 14 after treatment (scale bar: 1 mm). Magnified views (scale bars: 200 μm and 50 μm) show detailed tissue structures. Red arrows indicate inflammatory cell infiltration, green arrows indicate hair follicles, and blue arrows indicate sebaceous glands. (**b**) Representative Masson’s trichrome staining images at days 7 and 14 (scale bar: 1 mm), with magnified images highlighting collagen deposition (scale bars: 200 μm and 50 μm). (**c**) Quantitative analysis of collagen volume fraction at day 7 and day 14 (*n* = 3). ** *p* < 0.01 , *** *p* < 0.001, ns denotes no significant difference.

Histological and immunohistochemical (IHC) analyses elucidated the cellular mechanisms behind the accelerated healing, and the IHC score enables quantitative analysis of stained tissue through a scoring system [48]. Hematoxylin and eosin (H&E) staining showed that on Day 7, wounds treated with CS-SH-H hydrogel exhibited less inflammatory cell infiltration and more advanced granulation tissue formation compared to other groups. By Day 14, these wounds presented with a more organized epidermal and dermal architecture, including signs of regenerated skin appendages (Figure 5a). Masson’s Trichrome staining (MTS) for collagen confirmed superior tissue remodeling. On Day 7, CS-SH-H-treated wounds already displayed more abundant and denser collagen deposition. By Day 14, the collagen fibers in this group were markedly thicker, more aligned, and resembled a mature, basket-weave pattern (Figure 5b,c), indicating an accelerated and higher-quality tissue repair process. These findings provide direct evidence that the HMFP-mediated network reinforcement does not merely accelerate the early inflammatory-to-proliferative transition but also substantially enhances the quality of the later proliferative and remodeling phases, thus fulfilling the objective of coordinated modulation of the wound healing cascade in vivo.

**Figure 6:**
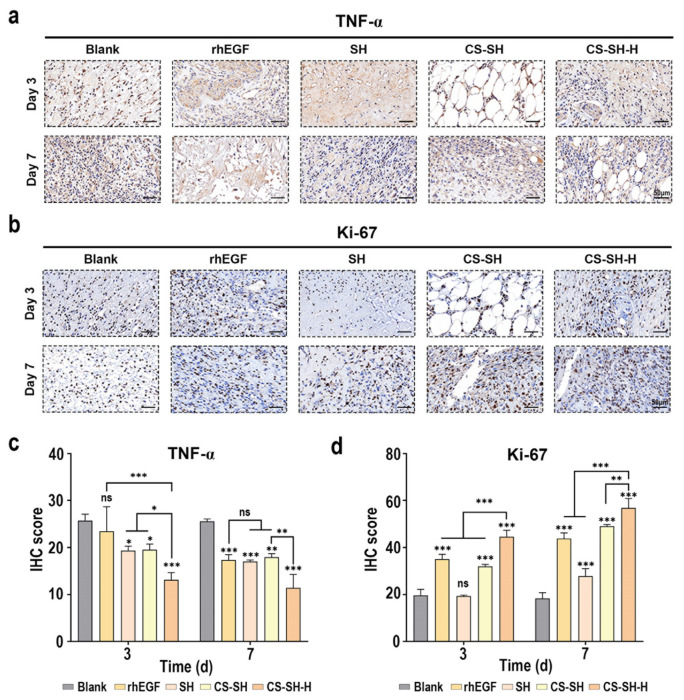
Immunohistochemical analysis of inflammation and cell proliferation markers in wound tissues. (**a**) Representative IHC staining images of TNF-α expression at days 3 and 7 after treatment (scale bar: 50 μm). (**b**) Representative immunohistochemical (IHC) staining images of Ki-67 expression at days 3 and 7 after treatment (scale bar: 50 μm). (**c**) Quantitative analysis of TNF-α IHC scores (*n* = 3). (**d**) Quantitative analysis of Ki-67 IHC scores (*n* = 3). * *p* < 0.05, ** *p* < 0.01, *** *p* < 0.001, ns denotes no significant difference.

IHC targeting inflammatory (TNF-α) and proliferative (Ki-67) markers provided molecular insight into this coordination. At Day 7, wounds treated with CS-SH-H hydrogel exhibited a 30% reduction in TNF-α expression compared to the positive rhEGF control and the untreated group, highlighting its potent anti-inflammatory effect (Figure 6a,c). Concurrently, the same tissue sections showed a 20% increase in Ki-67-positive cells (Figure 6b,d), indicative of enhanced cellular proliferation. In contrast, the base CS-SH hydrogel exhibited a weaker, less-coordinated effect on these markers. Collectively, these data provide compelling in vivo evidence that the HMFP-modified hydrogel actively coordinates the wound healing cascade by simultaneously dampening detrimental inflammation and promoting proliferative tissue repair, thereby accelerating functional tissue regeneration. This co-suppression of pro-inflammatory signaling (TNF-α) and co-enhancement of proliferative activity (Ki-67) is not a simple additive effect. Based on our previous investigations demonstrating the immunomodulatory activities of HMFP, it is plausible that preservation of these intrinsic bioactivities following hydrogel incorporation may contribute to the accelerated wound healing observed in the present study. However, direct validation of these signaling events within the hydrogel-treated wound microenvironment warrants further investigation. This hypothesis aligns with our previous work and warrants detailed molecular investigations in the future [41].

The present study delineates a distinct departure from conventional approaches in which biofunctionality is “added” to a hydrogel post-fabrication. Instead, we present a model where bioactive regulation is intrinsically engineered into the network’s fabric by using the HMFP itself as a structural modifier. Our approach thereby bridges network stability and therapeutic bioactivity.

Nevertheless, this study still has certain limitations. First, while we demonstrate the efficacy of HMFP from *Mytilus edulis*, its universality across other marine-derived adhesive or structural proteins (e.g., from barnacles, squids, or sea cucumbers) remains unexplored. A systematic comparative study is warranted to establish whether this “network regulator” principle is a unique property of HMFP or a more generalizable strategy for designing bioactive hydrogels using marine biopolymers. Second, the precise spatiotemporal dynamics of HMFP release or presentation from the hydrogel network in the wound bed, and its subsequent intracellular signaling cascades, require detailed pharmacokinetic and mechanistic elucidation in animal models. Third, the characterization of hydrogel properties in this study is rather preliminary. The mechanical properties of the hydrogel in a dynamic, hydrated state, which are crucial for dressing compliance and tissue engineering, should be comprehensively profiled.

Despite these limitations, the evidence strongly supports our core proposition. We propose HMFP, and potentially similar marine proteins, not merely as adhesives or bioactive factors but as a regulator of network organization and structural stabilization. This new biomaterial design paradigm, leveraging the native physicochemical and biological attributes of marine biopolymers to simultaneously dictate material self-assembly and biological outcome, opens vast possibilities for developing the next generation of smart, nature-inspired regenerative implants. Our results provide a strong foundation for this concept and pave the way for further optimization and broader application in skin regeneration, nerve repair, and other areas of tissue engineering.

## 3. Conclusions

**Figure 7:**
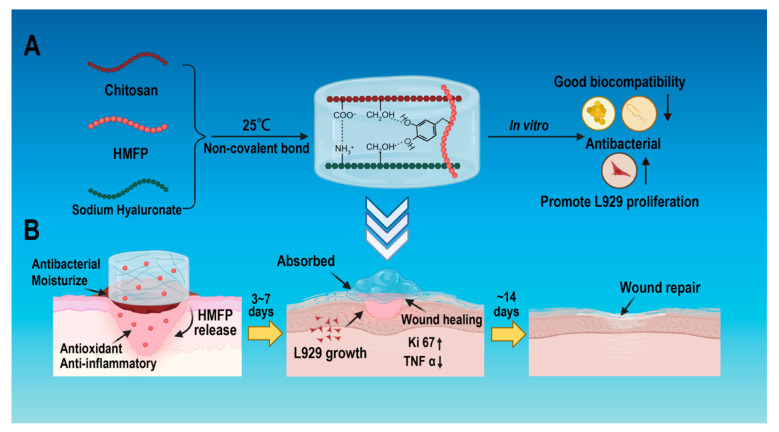
(**A**,**B**) Schematic illustration of CS-SH-H hydrogel fabrication and its proposed mechanism in promoting wound healing in a mouse full-thickness skin defect model. The schematic depicts the preparation process of the CS-SH-H hydrogel, the local microenvironment formed at the wound site, and the integrated effects of HMFP-mediated network regulation on antibacterial activity, inflammation modulation, and tissue regeneration during wound repair.

In conclusion, we have engineered a β-chitosan (CS)/sodium hyaluronate (SH) composite hydrogel (CS-SH-H) that exhibits synergistic enhancement in structure, function, and in vivo wound-healing efficacy (Figure 7). This study demonstrates a paradigm shift in biomaterial design, moving beyond simple additive blending to develop an integrative “network regulator” strategy. By incorporating a highly soluble mussel foot protein (HMFP) into the β-chitosan (CS) and sodium hyaluronate (SH) matrix, we fabricated a dual-functional hydrogel (CS-SH-H). HMFP serves as both a structural crosslinker, refining the pore architecture and enhancing stability via multivalent interactions, and a bioactive effector. The composite hydrogel synergistically couples preserved antibacterial activity with significantly enhanced cytocompatibility, promoting fibroblast functions. In a mouse full-thickness wound model, CS-SH-H hydrogel dramatically accelerated wound closure and tissue remodeling. This superior healing was mechanistically driven by HMFP-mediated orchestrated regulation- simultaneously dampening TNF-α-mediated inflammation and boosting Ki-67+ proliferative cells. Therefore, this work establishes marine-derived HMFP as a promising scaffold-functionalizing biopolymer to overcome the stability-activity trade-off in polysaccharide hydrogels, providing a scalable and synergistic platform for treating inflammatory and infected wounds.

## 4. Materials and Methods

### 4.1. Materials and Cell Line

Chitosan (CS, from squid gladius, DD > 95%) and the highly soluble mussel foot protein (HMFP) were obtained according to previously reported methods [33,49]. All reagents used in the purification of CS and the fabrication of HMFP were of analytical grade or above and were used without further purification. Sodium hyaluronate (SH, Mw ≈ 1.5 × 106 Da, pharmaceutical grade) was sourced from Beijing Wokai Biotechnology Co. Ltd. (Beijing, China). Acetic acid (glacial, ≥99.8%), phosphate-buffered saline (PBS, pH 7.4, 1X), and other general chemical reagents were purchased from Sinopharm Chemical Reagent Co., Ltd. (Shanghai, China). Antibacterial test materials, including Luria–Bertani (LB) agar and broth, were purchased from Haibo Biotech (Qingdao, China). For cell culture, Dulbecco’s Modified Eagle Medium (DMEM, high glucose), fetal bovine serum (FBS), and penicillin-streptomycin (P/S, 100×) solution were obtained from Gibco (Thermo Fisher Scientific, Waltham, MA, USA). The Cell Counting Kit-8 (CCK-8) and Calcein-AM/propidium iodide (Calcein-AM/PI) Live/Dead Double Staining Kit were procured from Beyotime Biotechnology (Shanghai, China).

The L929 mouse fibroblast cell line was obtained from the Cell Bank of the Chinese Academy of Sciences (Shanghai, China) and cultured in DMEM supplemented with 10% (*v*/*v*) FBS and 1% (*v*/*v*) Penicillin/Streptomycin. Cells were maintained in a humidified incubator at 37 °C under 5% CO_2_ and passaged when reaching 80–90% confluency. All cells used in experiments were within passages 3–5 and were tested routinely for mycoplasma contamination.

### 4.2. Preparation of Hydrogels

CS and SH stock solutions were prepared separately. Briefly, CS was dissolved in 0.5% (*v*/*v*) aqueous acetic acid solution under gentle magnetic stirring (500 rpm) at 25 °C for 2 h to achieve a clear and homogeneous 1.5% (*w*/*v*) solution. SH was dissolved in deionized water under identical stirring conditions (500 rpm, 25 °C) for 2 h to yield a 1.5% (*w*/*v*) solution.

#### 4.2.1. Fabrication of the Base (CS-SH) Hydrogel

The transparent CS solution was cooled to room temperature (RT, 25 °C), and the SH solution was similarly cooled. Subsequently, the CS and SH solutions were mixed at a volume ratio of 1:2 (CS:SH) under gentle vortexing to ensure instantaneous gelation. The mixture was immediately transferred to the desired polystyrene molds and allowed to stand undisturbed at RT for 30 min, forming the base β-chitosan/sodium hyaluronate (CS-SH) hydrogel.

#### 4.2.2. Fabrication of the Composite (CS-SH-H) Hydrogel

Prior to mixing, a 0.5 mg/mL stock solution of HMFP was freshly prepared in PBS (pH 7.4) and sterilized through a 0.22-μm pore-size filter. To prepare the composite hydrogel (CS-SH-H), a predetermined volume of the HMFP stock solution was first pre-mixed with the 1.5% CS solution, achieving a final HMFP concentration of 0.3 mg/mL in the combined CS/HMFP phase. This mixture was then combined with the SH solution at the 1:2 (CS:SH) ratio as described above. The gelation kinetics were unaltered, with bulk hydrogel formation occurring within 30 min at RT. For all experiments, representative hydrogel samples from at least three independent fabrication batches were used.

### 4.3. Morphological and Physicochemical Characterizations

#### 4.3.1. Scanning Electron Microscopy (SEM)

SEM was utilized to examine the three-dimensional microstructure of the hydrogels. The pre-fabricated hydrogels were rapidly frozen in liquid nitrogen for 10 min, followed by lyophilization (GeminiSEM 300, Carl Zeiss, Jena, Germany) for 48 h. The lyophilized samples were then mounted on conductive carbon tape, sputter-coated with a 10 nm gold layer (SC7620, Quorum Technologies, Laughton, UK), and imaged by field-emission SEM at 3.0 kV with a working distance of 8.8 mm. Representative fields per sample were captured at magnifications ranging from 100×.

#### 4.3.2. Fourier-Transform Infrared (FTIR) Spectroscopy

Chemical composition and intermolecular interactions within the hydrogels were analyzed using attenuated total reflection FTIR. Lyophilized hydrogel powders (*n* = 3) were directly placed on the diamond crystal of an FTIR spectrometer (Nicolet iS20, Thermo Fisher Scientific, Waltham, MA, USA). Spectra were collected over a wavenumber range of 400 to 4000 cm^−1^ with a resolution of 4 cm^−1^ and 128 accumulated scans per sample. Baseline correction and peak analysis were performed using OMNIC 9.2 software (Thermo Fisher Scientific, Waltham, MA, USA).

#### 4.3.3. Thermogravimetric Analysis (TGA)

Thermal stability and decomposition profiles were evaluated by TGA. Approximately 5.0 ± 0.1 mg of lyophilized hydrogel samples (*n* = 3) were loaded into alumina crucibles and heated from 25 °C to 800 °C at a constant rate of 10 °C min^−1^ under a continuous N_2_ flow of 40 mL min^−1^ in a TGA instrument (TG 209 F1 Libra, NETZSCH, Selb, Germany). The mass loss (TG) and its derivative (DTG) curves were recorded and analyzed.

### 4.4. Hydrogel Stability and pH Assessment

The physiological stability of the hydrogels was quantified via a mass retention assay. Hydrogels (approximately 2.0 g) were cast in 35 mm Petri dishes, and their initial mass was recorded after setting (m_1_). Samples were then submerged in 5 mL of PBS (pH 7.4) and incubated at 37 °C in a shaking incubator (100 rpm). At predetermined time points (3, 6, 9, 12, and 24 h), the samples were gently wiped with filter paper to eliminate excess liquid on the surface, followed by reweighing. (m_t_). The residual hydrogel percentage (%) was calculated as: Residual mass (%) = (m_t_ − m_0_)/(m_1_ − m_0_) × 100%, where m_0_ is the mass of the empty dish.

pH values of hydrogel extracts were measured after incubating 1 g of hydrogel in 10 mL PBS at 37 °C for 48 h, using a calibrated pH meter (Mettler Toledo, Zurich, Switzerland). Measurements were repeated (*n* = 6).

### 4.5. In Vitro Cytocompatibility and Cell Functional Assays

All in vitro experiments utilized L929 fibroblasts (1 × 10^4^ cells/well for 96-well plates, 5 × 10^4^ cells/well for 24-well plates) seeded one day prior to treatment.

#### 4.5.1. Biocompatibility via Hydrogel Extracts

Hydrogel extracts were prepared by submerging 1 g of sterile hydrogel in 10 mL of complete DMEM medium and incubating at 37 °C for 24 h with gentle agitation (50 rpm). The supernatant was collected, sterile-filtered (0.22 µm), and diluted with fresh medium. Cells were incubated with extracts for 24 h before performing the CCK-8 assay according to the manufacturer’s protocol. Absorbance at 450 nm was measured using a microplate reader (Synergy H1, BioTek, Winooski, VT, USA). Cell viability was expressed as a percentage relative to the control group (cells cultured in fresh medium only, defined as 100%).

#### 4.5.2. HMFP Bioactivity (Direct Cell Assays)

To assess the intrinsic bioactivity of free HMFP, cells were treated with a dilution series of HMFP (1 × 10^−2^ to 1 × 10^−5^ mg/mL prepared in complete medium).

Cell proliferation (CCK-8): Cell viability/proliferation was measured at 24 and 48 h post-treatment.

Cell migration (Scratch assay): A linear scratch wound was generated on a confluent cell monolayer by means of a sterile 200 µL pipette tip. Detached cells were washed away with PBS, and fresh medium containing HMFP (1 × 10^−3^, 1 × 10^−4^, 1 × and 10^−5^ mg/mL) or vehicle control (serum-free medium) was added. Scratch closure was monitored at 0, 12, and 24 h using an inverted phase-contrast microscope (Nikon Eclipse Ts2, Nikon, Toyko, Japan). The percentage of wound closure was quantified using ImageJ 1.80p software.

Live/dead staining: After 24 h of HMFP treatment, cells were stained with the Calcein-AM/PI dual-staining kit following the manufacturer’s instructions and visualized using a fluorescence microscope (Nikon, Japan). Viable (green) and dead (red) cells were quantified from three random fields per well (*n* = 3).

### 4.6. Antibacterial Activity Evaluation

The antibacterial efficacy of the hydrogels against representative Gram-negative (*Escherichia coli*, ATCC 25922) and Gram-positive (*Staphylococcus aureus*, ATCC 6538) bacteria was evaluated. Overnight bacterial cultures were diluted to a density of 1 × 10^7^ colony-forming units (CFU) per mL in sterile PBS. Sterilized hydrogel discs (10 mm diameter, about 2 mm thickness) were placed in a 6-well plate, and 2 mL of bacterial suspension was added to completely cover the hydrogel. A PBS-only group served as the negative control. At selected time points (0, 1, 2 h), 100 µL aliquots were drawn from the bacterial suspension, serially diluted (10-fold), and applied to LB agar plates. After incubation at 37 °C overnight, the number of bacterial colonies was determined. The antibacterial inhibition rate (%) was calculated as: Inhibition Rate (%) = (CFUcontrol − CFUtest)/CFUcontrol × 100%. Experiments were performed in biological triplicate (*n* = 3).

### 4.7. Hemocompatibility Assessment

Fresh rat whole blood (anticoagulated with heparin) was centrifuged at 1000× *g* for 10 min at 4 °C to obtain red blood cells (RBCs). The RBCs were then washed three times with PBS and resuspended to produce a 4% (*v*/*v*) RBC suspension. A total of 500 µL of the RBC suspension was mixed with 500 µL of hydrogel extracts (prepared as in Section 4.5.1). PBS and 0.5% Triton X-100 were used as the negative control (A_n_) and positive control (A_p_). Following incubation at 37 °C for 1 h, the samples were centrifuged at 1000× *g* for 5 min. The absorbance of the supernatant at 540 nm (As) was measured. The hemolysis ratio (%) was calculated using the formula: Hemolysis (%) = (A_s_ − A_n_)/(A_p_ − A_n_) × 100%. All assays were conducted in triplicate (*n* = 3). A hemolysis ratio below 5% was deemed acceptable for biomaterials.

### 4.8. In Vivo Wound Healing Study in a Mouse Model

All animal procedures were approved by the Institutional Animal Care and Use Committee (IACUC) of Shanghai Ocean University (Approval No. SHOU-DW-2025-160) and conducted in accordance with ARRIVE guidelines. Specific pathogen-free (SPF) male Kunming mice (8 weeks old, 30–35 g) were acclimatized for one week under standard conditions (12/12 h light/dark cycle, 25 ± 2 °C, 55 ± 10% humidity, ad libitum access to food and water).

#### 4.8.1. Surgical Procedure

Mice were anesthetized via intraperitoneal injection of sodium pentobarbital (50 mg/kg). Dorsal hair was removed, and the skin was sterilized with iodophor. A single, full-thickness excisional wound (8 mm in diameter) was created on the dorsal region using sterile surgical scissors. The panniculus carnosus muscle layer was carefully removed.

#### 4.8.2. Treatment Groups

Mice were randomly assigned to five experimental groups (*n* = 3 per group):

Untreated group (Negative Control): Wounds were left uncovered. Positive Control group: Treated topically with recombinant human epidermal growth factor gel from Guilin Pavay Gene Pharmaceutical Co. Ltd. (Guilin, China). SH group: Treated with 1.5% SH solution. CS-SH group: Treated with base hydrogel. CS-SH-H group: Treated with HMFP-integrated composite hydrogel.

Hydrogels (100 µL, covering the entire wound bed) were applied every 3–4 days, and digital photographs of the wounds were taken on days 0, 3, 7, and 14 using a digital camera. Wound areas were quantified using ImageJ 1.80p software, and the percentage of wound closure was calculated relative to the initial area (day 0).

### 4.9. Histological and Immunohistochemical (IHC) Analyses

On days 7 and 14 post-surgery, mice were euthanized, and wound tissue including 2 mm of surrounding normal skin was collected.

#### 4.9.1. Histology

Tissues were subjected to fixation in 4% (*v*/*v*) paraformaldehyde for 24 h at 4 °C, followed by paraffin embedding and sectioning into 5-µm slices. Sections were stained with Hematoxylin and Eosin (H&E) and Masson’s Trichrome staining (MTS) for general histology and collagen deposition assessment, respectively, following standard protocols.

#### 4.9.2. Immunohistochemistry

Paraffin sections were subjected to antigen retrieval in citrate buffer (pH 6.0) at 95 °C for 15 min, followed by blocking of endogenous peroxidase with 3% H_2_O_2_ and nonspecific binding with 5% bovine serum albumin (BSA). Sections were incubated overnight at 4 °C with the following primary antibodies diluted in antibody diluent: Rabbit anti-Ki-67 (1:200, Abcam, Cambridge, UK, ab16667) and Rabbit anti-TNF-α (1:150, Abcam, Cambridge, UK, ab6671). After washing, HRP-conjugated goat anti-rabbit IgG secondary antibody (1:500) was added for 1 h at RT, followed by development with 3,3′-diaminobenzidine (DAB) substrate and counterstaining with hematoxylin.

All stained sections were imaged using a light microscope (Olympus BX53, Tokyo, Japan). Quantitative analysis of the percentage of positive cells (Ki-67) or the area of positive staining (TNF-α) was performed using ImageJ 1.80p software for at least five random high-power fields (400× magnification) per sample.

### 4.10. Statistical Analysis

All quantitative data are presented as mean ± standard deviation (SD) from at least three independent experiments. Statistical analysis was performed using GraphPad Prism software (v10.0). Normality of data distribution was confirmed using the Shapiro–Wilk test. For multiple-group comparisons, one-way analysis of variance (ANOVA) followed by Duncan’s multiple-range post hoc test was used. For time-course data, two-way ANOVA with Tukey’s post hoc test was applied. 

## Data Availability

The data that support the findings of this study are available from the corresponding author upon reasonable request.

## Abbreviations

The following abbreviations are used in this manuscript:

HMFP	Highly soluble mussel foot protein
CS	β-Chitosan
SH	Sodium hyaluronate
MAPs	Mussel adhesive proteins
SEM	scanning electron microscopy
FTIR	Fourier-transform infrared
TGA	Thermogravimetric analysis
DTG	Derivative thermogravimetric
rhEGF	Recombinant human epidermal growth factor
*E. coli*	*Escherichia coli*
*S. aureus*	*Staphylococcus aureus*
